# Intraductal fibroadenoma presenting with nipple bleeding in an adolescent: a case report and literature review

**DOI:** 10.3389/fped.2025.1661307

**Published:** 2025-11-14

**Authors:** Pingfa Gao, Wenyan Yang, Wenbin Guan, Ping Wu, Wenjie Lv

**Affiliations:** 1 Department of Thyroid and Breast Surgery, Chongming Hospital Affiliated to Shanghai University of Medicine and Health Sciences, Shanghai, China; 2 Shanghai University of Medicine and Health Sciences, Shanghai, China; 3 Department of Pathology, Xinhua Hospital Affiliated to Shanghai Jiao Tong University School of Medicine, Shanghai, China; 4 Department of Breast Surgery, Xinhua Hospital Affiliated to Shanghai Jiao Tong University School of Medicine, Shanghai, China

**Keywords:** intraductal fibroadenoma, adolescent breast lesions, nipple discharge, stromal infarction, benign breast tumors

## Abstract

A 12-year-old Chinese adolescent girl presented with a 2-month history of left nipple bleeding. Physical examination revealed bloody and brownish nipple discharge. Ultrasonography identified a 1 mm solid mass within a dilated duct, and MRI showed heterogeneous enhancement. Surgical exploration revealed a well-defined, medium-textured mass within the duct, which was completely excised. Histopathological analysis confirmed an intraductal fibroadenoma with stromal infarction and hemorrhage, explaining the nipple discharge. Postoperatively, the patient recovered well without recurrence during a 12-month follow-up. Fibroadenoma, the most common benign breast lesion in adolescents, arises from estrogen-sensitive intralobular fibroblasts and may involve ductal structures, as in this case. Spontaneous infarction of intraductal fibroadenoma is exceptionally rare and can present with bloody discharge, necessitating differentiation from other intraductal lesions such as ductal adenoma and intraductal papilloma. This case highlights the importance of timely diagnosis and management of breast lesions in adolescents, emphasizing the need to distinguish between physiological changes and pathological conditions. The report also discusses the etiology of spontaneous infarction in intraductal fibroadenoma and its clinical implications, contributing to the understanding of this rare presentation in pediatric patients.

## Introduction

During adolescence, the female breast undergoes various changes influenced by genetic, nutritional, endocrine, and other factors (1). These breast changes in adolescent patients can be either physiological or pathological, highlighting the critical importance of timely detection and identification of malignant lesions. The incidence of breast diseases is relatively low in adolescent girls, with fibroadenoma being the most common condition (2). Fibroadenoma is a benign lesion composed of both epithelial and stromal components. It arises from an abnormal increase in the sensitivity of intralobular fibroblasts to estrogen, which may be related to abnormalities in the quantity or quality of estrogen receptors within these fibroblasts (3). When fibroadenoma occurs within the duct, it is referred to as intraductal fibroadenoma. This condition holds significant diagnostic importance regarding symptoms and pathology, necessitating differentiation from other conditions such as ductal adenoma and intraductal papilloma. Spontaneous infarction of intraductal fibroadenoma is an exceptionally rare finding in recent literature.

In this case report, we present a 12-year-old girl who presented with bloody nipple discharge from the left breast. Imaging studies revealed an intraductal mass, which was subsequently managed surgically. Postoperative pathology confirmed the diagnosis of intraductal fibroadenoma with infarction, elucidating the clinical symptom of bloody discharge. Furthermore, this article explores the differential diagnosis of benign intraductal lesions and discusses the potential etiologies and clinical manifestations of spontaneous infarction in intraductal fibroadenoma.

## Case presentation

**Figure 1:**
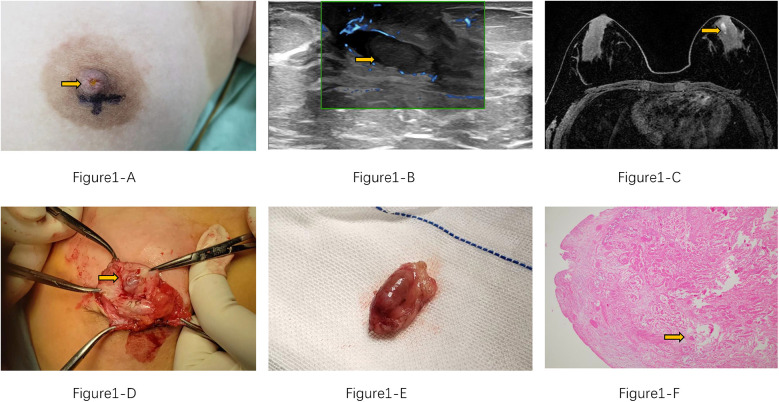
**(A)** Brownish discharge from the patient's left nipple (yellow arrow). **(B)** Ultrasound shows a dilated duct with an intraluminal mass (yellow arrow). **(C)** MRI reveals ductal dilation with a filling defect (yellow arrow). **(D)** Intraoperative view of the intraluminal mass within the duct. **(E)** Complete surgical excision of the mass. **(F)** HE*40 histopathological section shows the tumor infarction is evident, with fibrin and red blood cells alternating faintly visible within the vascular lumen. (yellow arrow).

The patient is a 12-year-old Chinese adolescent female who presented with a two-month history of nipple discharge, which had evolved from pale yellow to brown in color, accompanied by intermittent breast distension and tenderness. Physical examination revealed no evidence of erythema, swelling, or ulceration on the skin surface. Upon manual compression of the left nipple, bloody discharge was elicited, and no palpable mass was identified (Figure 1A). The patient experienced menarche at 11 years of age and has maintained regular menstrual cycles. Her personal history is negative for smoking, alcohol use, and substance abuse. There is no family history of genetic or hereditary disorders, and she has no significant history of specific environmental or occupational exposures.

Post-admission ultrasound revealed localized dilation of the left breast duct posterior to the nipple, with a maximum internal diameter of 5.2 mm. A linear hypoechoic lesion measuring approximately 13 mm × 5 mm was identified within the dilated duct (Figure 1B). The lesion exhibited a regular morphology, parallel orientation, and heterogeneous echogenicity, suggestive of intraductal papilloma, and was classified as BI-RADS Category 3. No significant lymphadenopathy was observed in the bilateral axillary regions. Magnetic resonance imaging (MRI) showed a linear hyperintense signal on T1-weighted imaging (T1WI) medial to the left nipple, with rim enhancement post-contrast. A small, round nodule (0.7 cm in diameter) was noted posteriorly (Figure 1C), displaying well-defined margins, isointensity on T2-weighted imaging (T2WI), and hyperintensity on diffusion-weighted imaging (DWI), with an apparent diffusion coefficient (ADC) value of 1.72 × 10^−3^ mm^2^/s. The lesion demonstrated linear enhancement and a moderate-rising time-intensity curve (TIC). It was provisionally classified as BI-RADS Category 3, with intraductal papilloma considered a differential diagnosis. No abnormal laboratory findings were noted preoperatively.

**Figure 2:**
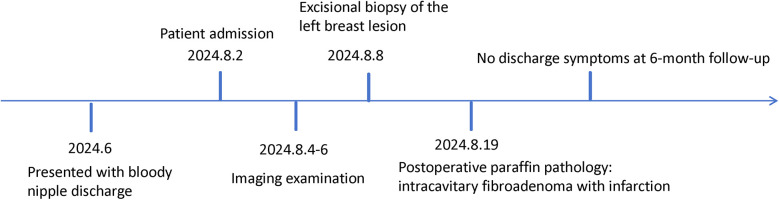
The timeline of the treatment.

Subsequently, the patient underwent surgical excision of the left breast lesion. Intraoperatively, yellowish discharge was observed from the left nipple, and a dilated duct with a palpable mass measuring 8 mm was noted posterior to the nipple. The mass had a moderate consistency and clear boundaries. The resected tissue was a grayish-white and grayish-red mass, measuring 20 × 15 × 10 mm (Figures 1D,E). Histopathological examination of the paraffin-embedded specimen revealed an intracanalicular fibroadenoma with infarction (Figure 1F). Figure 2 demonstrates the patient's treatment timeline. The patient was followed up at our institution every three months postoperatively. No recurrence of nipple discharge was observed during the one-year follow-up period.

## Discussion

Intraductal fibroadenoma is an exceptionally rare condition, with only 14 cases reported in the literature. It predominantly affects adolescent females, particularly those aged 15 years or younger (4, 5), though a small number of cases have been documented in patients aged 30 to 50 years (6, 7). Occurrences in elderly patients are exceedingly rare (8). Postmenarchal adolescent females are consistently identified as the high-risk group for this condition (4, 9–11).

In younger patients, intraductal fibroadenoma often exhibits complex pathological features, overlapping with papilloma, intraductal adenoma, fibroadenoma, and benign phyllodes tumor (6, 12). In contrast, fibroadenomas in older, perimenopausal women are typically solitary, grow more slowly, and carry a marginally increased risk of associated pathology, such as sclerosing adenosis or carcinoma *in situ* within complex fibroadenomas, often prompting a lower threshold for diagnostic excision to rule out malignancy. The etiology is thought to mirror that of conventional fibroadenoma, potentially linked to hormonal imbalances involving estrogen and progesterone, as well as abnormalities in estrogen receptor functionality in fibroblasts (9, 13). Clinically, adolescent females typically present with a rapidly enlarging, palpable breast mass over several months, sometimes accompanied by bloody nipple discharge and/or breast pain. Imaging studies usually reveal a well-circumscribed, mildly lobulated, heterogeneous mass with uniform density (4, 6). In cases of malignant transformation, imaging may show heterogeneous density, irregular margins, and reduced apparent diffusion coefficient (ADC) values (14).

Breast fibroadenomas can be classified into three distinct types: simple fibroadenoma, juvenile fibroadenoma, and multicentric fibroadenoma. Among these, simple fibroadenoma is the most prevalent, characterized by small tumor size and slow growth. Juvenile fibroadenoma, which has the lowest incidence, typically develops after menarche and exhibits rapid growth. A rare subtype of juvenile fibroadenoma is giant juvenile fibroadenoma (3). Intraductal fibroadenoma may follow a similar classification; however, it is less frequently described in the current literature.

Histologically, intraductal fibroadenoma is characterized by fibroadenomatous polypoid structures growing within ducts or multiple polyps resembling papillary hyperplasia, often filling the ductal lumen (6, 12). It typically presents as solitary or multiple adenomatous nodules, featuring proliferative tubular myoepithelium with luminal and basal cells surrounded by fibrous stroma (15). Differentiation from other intraductal lesions, such as ductal adenoma and intraductal papilloma, is essential. Ductal adenoma, a benign lesion confined to the ductal lumen, is distinguished by well-demarcated margins, duct wall thickening, fibrosis, or plaque-like calcification, often with abundant elastic tissue. Its solid component consists of glandular structures within a hyalinized stroma, composed of biphasic epithelial and myoepithelial cells. Epithelial cells exhibit cuboidal to columnar morphology with intense eosinophilic staining, while myoepithelial cells are smaller and cuboidal to flattened, with clear or faintly eosinophilic staining. A key feature is apical cytoplasmic snouting in epithelial cells at the glandular lumen interface (16, 17). Intraductal papillomas, more common in perimenopausal women, feature ductal dilation and a complex fibrovascular core lined by myoepithelial and luminal epithelial cells. Multicentric peripheral papillomas carry a higher risk of malignant transformation (18, 19). Intraductal fibroadenomas often share overlapping features with papillomas, adenomas, fibroadenomas, and benign phyllodes tumors, complicating diagnosis (6, 12). Table 1 summarizes these differential diagnostic features.

**Table 1:** A comprehensive comparison of clinical, pathological, and diagnostic features among duct adenoma, intraductal papilloma, and intraductal fibroadenoma, highlighting key distinguishing points for clinical practice.

Feature/type	Duct adenoma	Intraductal papilloma	Intraductal fibroadenoma
Tissue Origin	Originates from the nipple duct opening or surrounding ducts	Originates from the central or peripheral mammary ducts	Originates from the terminal duct lobular unit
Gross Morphology	Well-defined mass, may involve the nipple skin, with a grayish-white and pale-yellow cut surface	Grayish-red nodules within grayish-white and pale-yellow breast tissue, hard and poorly defined	Well-defined oval nodules, usually <3 cm, with a grayish-white, solid, rubbery, whorled cut surface, often with slit-like spaces
Microscopic Features	Proliferation of glandular epithelium, which may show papillary, adenosis, or sclerosing pseudo-infiltrative patterns, with myoepithelial cells surrounding the glands	Intraductal papillary proliferation with fibrovascular cores, outer luminal cells, and inner myoepithelial cells; papillary structures may be simple or complex	Biphasic tumor composed of epithelial and mesenchymal components; stromal cells are uniform without atypia, and epithelial components may show various morphological changes
Immunohistochemistry	Myoepithelial markers positive, CK5/14 and ER/PR show heterogeneous expression	Myoepithelial markers such as p63 can demonstrate the presence of myoepithelial cells	Presence of MED12 exon 2 mutation in the stromal component
Clinical Presentation	Often involves the nipple, may present as nipple erythema, erosion, or bloody discharge	May cause clear or bloody nipple discharge, usually painless	Typically presents as a painless, solitary, firm, slow-growing, mobile, well-defined nodule; rarely associated with bloody discharge
Typical Age Group	Middle-aged to older women (40–60 years)	Wide range (30–70 years), central papillomas often in perimenopausal women	Younger women (15–35 years), but can occur at any age
Imaging Findings (Ultrasound)	Solid, well-circumscribed, hypoechoic mass near the nipple; may show posterior acoustic enhancement	Dilated duct with an intraductal solid component; vascularity on Doppler	Homogeneous, hypoechoic, oval mass with circumscribed margins; may show posterior acoustic enhancement
Key Diagnostic Challenge	Differentiating from syringomatous adenoma and low-grade adenosquamous carcinoma	Distinguishing from papillary DCIS and invasive papillary carcinoma	Differentiating from phyllodes tumor (especially cellular variants)
Risk of Malignancy	Very low; associated with sclerosing adenosis patterns	Slightly increased risk (atypical papilloma carries higher risk)	Extremely low; malignant transformation is rare
Management Approach	Complete local excision for diagnosis and symptom relief	Surgical excision (especially for central/solitary papillomas with symptoms or atypia)	Active surveillance for typical lesions; excision for large, growing, or symptomatic tumors

The specific mechanisms underlying the development of intraductal fibroadenoma have been scarcely explored in the literature, and the precise etiology remains poorly understood. Current hypotheses suggest that the formation of intraductal fibroadenoma is primarily driven by the hyperplasia of stromal and connective tissues. The connective tissue frequently infiltrates the ductal wall, with stromal cells originating from the periductal and perilobular connective tissue. When the base of the protruding stroma is broad or expansive, it exerts compressive forces on the opposing ductal wall, leading to its eventual narrowing and the subsequent formation of a fibroadenoma (12, 20, 21).

Spontaneous infarction of fibroadenomas is a rare phenomenon, occurring in only 0.5%–1.5% of cases (11, 22), with intraductal fibroadenomas exhibiting this feature even more infrequently. Clinically, it often manifests as rapid enlargement of a pre-existing breast mass over a short period, accompanied by severe pain and, in some cases, bloody nipple discharge. Spontaneous infarction can occur in various benign breast lesions, such as fibroadenomas, intraductal papillomas, and phyllodes tumors, particularly during phases of hormonal fluctuation. Bloody nipple discharge may also arise when benign lesions involve the areolar region or ducts (10).

The mechanisms underlying spontaneous infarction in intraductal fibroadenomas are attributed to both physiological and iatrogenic factors. Physiological factors include degenerative changes, such as calcification and hyaline degeneration, and ischemic events within hyperplastic tumor tissues. Iatrogenic factors, such as trauma or fine-needle aspiration, can induce thrombotic vascular changes, while hormonal imbalances from oral medications may also contribute (5). Histologically, infarction in fibroadenomas often presents as a solitary cyst with yellow-red vegetations and central necrosis. For intraductal fibroadenomas, pre-treatment evaluation should include culture of ductal secretions, measurement of estrogen and progesterone levels, and ultrasound imaging (10). Although ultrasound has limited sensitivity for detecting intratumoral infarction, it remains a valuable diagnostic tool and a critical basis for clinical diagnosis.

**Figure 3:**
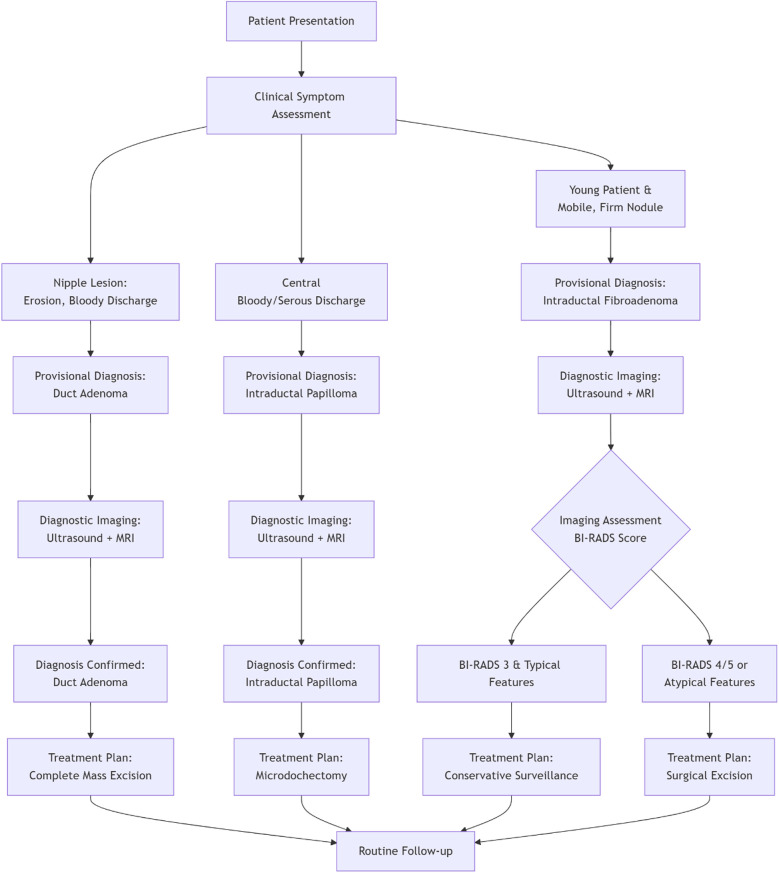
Clinical decision pathway for the diagnosis and management of common intraductal breast lesions.

The treatment of intraductal fibroadenoma in adolescents, as reported in the majority of literature, involves local surgical excision of the tumor (4–6, 11, 12). Follow-up at 6 or 24 months postoperatively has shown no recurrence or related complications in these cases. In the present case, the patient underwent simple mass excision, and no recurrence of nipple discharge was observed during the 12-month follow-up period. These findings demonstrate that intraductal fibroadenoma, as a benign condition, has an excellent prognosis following complete surgical resection of the lesion. Figure 3 provides a clear clinical pathway for differential diagnosis and management of three common breast lesions based on their characteristic presentations, imaging findings, and standardized assessment criteria.

The patient's postoperative course was uneventful; quarterly follow-up examinations at our institution for one year confirmed no recurrence of nipple discharge. While this affirms the short-term efficacy of the intervention, it underscores the necessity of a structured long-term follow-up strategy to ensure ongoing patient well-being.

In summary, this case highlights a rare instance of intraductal fibroadenoma with spontaneous infarction in an adolescent female, presenting with bloody nipple discharge. The surgical approach should be guided by comprehensive preoperative diagnosis and evaluation. For such lesions, thorough differential diagnosis both preoperatively and intraoperatively is crucial. In adolescent females, to minimize impact on normal breast development and future lactation, simple mass excision is preferred over regional duct excision for confirmed intraductal fibroadenomas. This strategy maximizes preservation of breast function and morphology.

## Data Availability

The original contributions presented in the study are included in the article/Supplementary Material, further inquiries can be directed to the corresponding author.
